# Supplementary material for: Stalagmite paleomagnetic record of a quiet mid-to-late Holocene field activity in central South America
Source: Nat Commun. 2022 Mar 15;13:1349. doi: 10.1038/s41467-022-28972-8 (PMC8924270; doi:10.1038/s41467-022-28972-8)
Supplement: Supplementary file 1 — Supplementary Information [file 41467_2022_28972_MOESM1_ESM.pdf]

Supplementary Information for "Stalagmite paleomagnetic record of a quiet mid-to-late Holocene field activity in central South America"

Supplementary Inventory:

Figures. S1 to S3

Table S1

References for SI reference citations

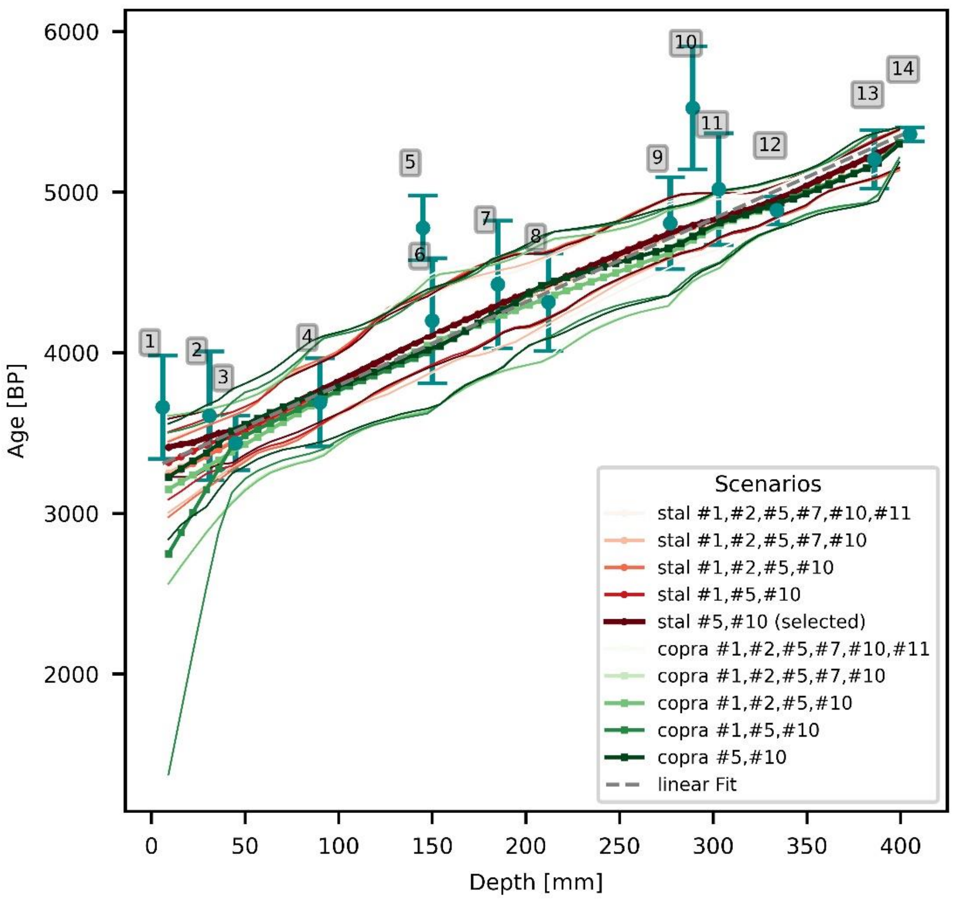

Supplementary Figure S1: Simulation of different age model scenarios by excluding points with median values reversed to their subsequent. These models were constructed using COPRA [1] and StalAge [2] algorithms. Points in cyan are the U-Th ages with their respective error bars.

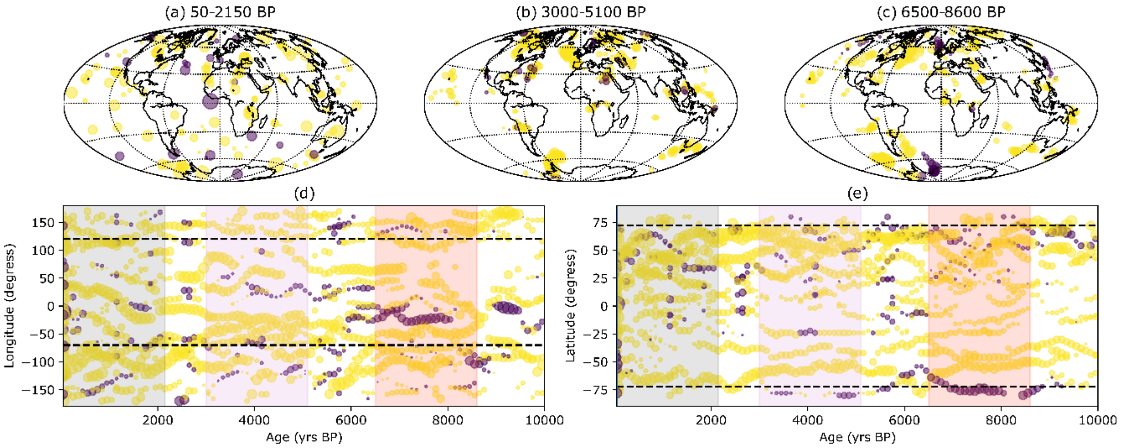

Supplementary Figure S2: Tracking of normal (NFP) and reverse (RFP) flux patches at the core mantle boundary for model HFM.OL1.A1 [3](a-c) the location of NFP (yellow) and RFP (purple) for time intervals #1 (a, 50-2150 BP), #2 (b, 3000-5100 BP), #3 (c, 6500-8600 BP). (d) longitudinal evolution of NFP (yellow circles) and RFP (purple circles); dashed lines indicate the azimuth of the positive peak of shear wave velocity in the mantle [4], rectangle shades correspond to interval #1 (light grey), #2 (light purple), #3 (light red) (e) latitudinal evolution of NFP (yellow circles) and RFP (purple circles); dashed lines indicate the expected latitude of the tangent cylinder in the northern and southern hemispheres. The size of purple and yellow circles is proportional to the intensity of the flux patch. Map generated using python package Cartopy.

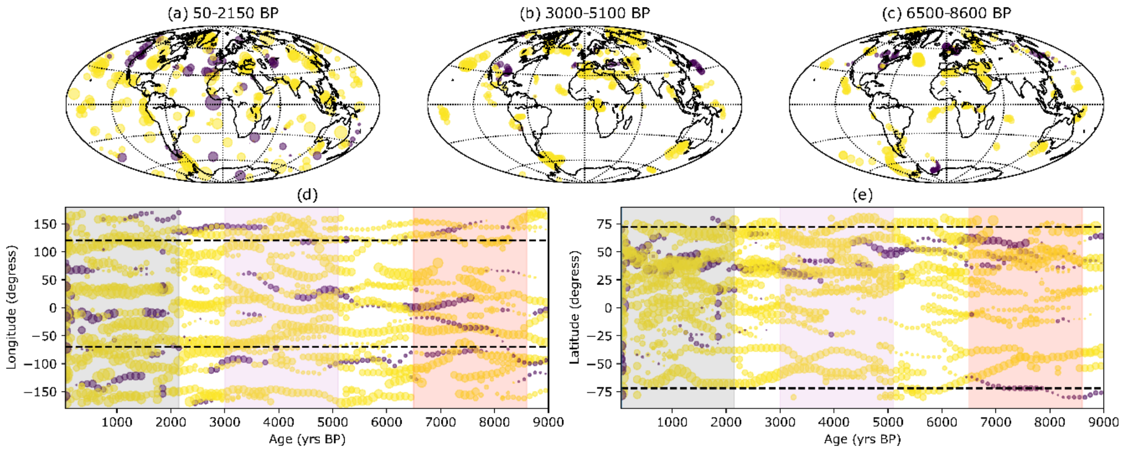

Supplementary Figure S3: Tracking of normal (NFP) and reverse (RFP) flux patches at the core mantle boundary for model pfm9k.1a [6](a-c) the location of NFP (yellow) and RFP (purple) for time intervals #1 (a, 50-2150 BP), #2 (b, 3000-5100 BP), #3 (c, 6500-8600 BP). (d) longitudinal evolution of NFP (yellow circles) and RFP (purple circles); dashed lines indicate the azimuth of the positive peak of shear wave velocity in the mantle (Masters et al., 2000), rectangle shades correspond to interval #1 (light grey), #2 (light purple), #3 (light red) (e) latitudinal evolution of NFP (yellow circles) and RFP (purple circles); dashed lines indicate the expected latitude of the tangent cylinder in the northern and southern hemispheres. The size of purple and yellow circles is proportional to the intensity of the flux patch. Map generated using python package Cartopy.

Table S1: ^{230}Th dating results. The error is 2σ error.

Sample Number	^{238}U (ppb)	^{232}Th (ppt)	^{230}Th / ^{232}Th (atomic x10^{-6})	δ^{234}U* (measured)	^{230}Th / ^{238}U (activity)	^{230}Th Age (yr) (uncorrected)	^{230}Th Age (yr) (corrected)	δ^{234}U$_{Initial}$** (corrected)	^{230}Th Age (yr BP)*** (corrected)
**DBE-50 6mm**	226.4 ±0.7	8806 ±178	22 ±1	264.9 ± 3.4	0.0526 ±0.0012	4625 ±110	**3729 ±643**	268 ±4	**3661 ±643**
**DBE-50 31mm**	269.0 ±0.7	13217 ±266	18 ±0	272.9 ± 3.6	0.0549 ±0.0009	4802 ±78	**3676 ±801**	276 ±4	**3608 ±801**
**DBE-50 45mm**	246.3 ±0.3	5108 ± 102	36 ±1	266.4 ± 1.6	0.0455 ±0.0003	3986 ±31	**3509 ±339**	269 ±2	**3439 ±339**
**DBE-50 90mm**	295.5 ±0.5	10060 ±202	26 ±1	294.3 ± 2.4	0.0527 ±0.0010	4525 ±88	**3759 ±549**	297 ±2	**3691 ±549**
**DBE-50 145mm**	372.5 ±2.2	9326 ±195	42 ±1	301.8 ± 5.6	0.0631 ±0.0009	5406 ±86	**4847 ±404**	306 ±6	**4778 ±404**
**DBE-50 150mm**	235.4 ±0.4	11428 ±229	21 ±1	300.8 ± 2.2	0.0624 ±0.0014	5355 ±126	**4267 ±780**	304 ±2	**4199 ±780**
**DBE-50 185mm**	296.5 ±0.5	14722 ± 295	22 ±0	286.9 ± 2.0	0.0648 ±0.0003	5620 ±32	**4495 ±797**	291 ±2	**4425 ±797**
**DBE-50 212mm**	389.3 ±1.1	14822 ±299	27 ±1	310.9 ± 3.4	0.0615 ±0.0011	5229 ±97	**4383 ±606**	315 ±4	**4315 ±606**
**DBE-50 277mm**	287.2 ±0.5	10281 ±206	30 ±1	303.7 ± 2.0	0.0662 ±0.0009	5673 ±81	**4874 ±571**	308 ±2	**4806 ±571**
**DBE-50 289mm**	274.4 ±0.6	12966 ±261	28 ±1	350.2 ± 3.6	0.0796 ±0.0031	6610 ±262	**5592 ±766**	356 ±4	**5524 ±766**
**DBE-50 303mm**	274.1 ±0.6	12245 ±246	27 ±1	345.9 ± 2.9	0.0728 ±0.0015	6053 ±132	**5087 ±696**	351 ±3	**5019 ±696**
**DBE-50 334mm**	789.0 ±1.1	8713 ± 175	94 ±2	350.6 ± 2.0	0.0629 ±0.0004	5194 ±36	**4956 ±172**	355 ±2	**4886 ±172**
**DBE-50 386mm**	285.5 ±0.9	6535 ±132	50 ±1	330.9 ± 4.2	0.0688 ±0.0010	5772 ±92	**5272 ±365**	336 ±4	**5204 ±365**
**DBE-50 405mm**	319.5 ±1.2	1258 ±26	268 ±6	293.4 ± 4.2	0.0639 ±0.0007	5519 ±61	**5430 ±87**	298 ±4	**5361 ±87**

U decay constants: λ_{238} = 1.55125x10^{-10} [7] and λ 234 = 2.82206x10^{-6} [8]. Th decay constant: λ_{230} = 9.1705x10-6 [8].
*δ^{234}U = ([^{234}U/^{238}U]$_{activity}$ – 1)x1000. ** δ^{234}U$_{initial}$ was calculated based on ^{230}Th age (T), i.e., δ^{234}U$_{initial}$ = δ^{234}U$_{measured}$ x $e^{\lambda_{234} \times T}$.
Corrected ^{230}Th ages assume the initial ^{230}Th/^{232}Th atomic ratio of 4.4 ±2.2 x10^{-6}. Those are the values for a material at secular equilibrium, with the bulk earth ^{232}Th/^{238}U value of 3.8. The errors are arbitrarily assumed to be 50%.
***B.P. stands for "Before Present" where the "Present" is defined as the year 1950 A.D.
Values in red denotes the points that were excluded from age modelling.

References

1 Breitenbach, S. F. M. *et al*. COnstructing Proxy Records from Age models (COPRA). *Climate of the Past* **8**, 1765-1779, doi:10.5194/cp-8-1765-2012 (2012).

2 Scholz, D. & Hoffmann, D. L. StalAge - An algorithm designed for construction of speleothem age models. *Quaternary Geochronology* **6**, 369-382, doi:10.1016/j.quageo.2011.02.002 (2011).

3 Constable, C., Korte, M. & Panovska, S. Persistent high paleosecular variation activity in southern hemisphere for at least 10 000 years. *Earth and Planetary Science Letters* **453**, 78-86, doi:10.1016/j.epsl.2016.08.015 (2016).

4 Masters, G., Laske, G., Bolton, H. & Dziewonski, A. in *Earth's Deep Interior: Mineral Physics and Tomography From the Atomic to the Global Scale* 63-87 (2000).

5 Office, M. (Met Office Exeter, United Kingdom, 2010).

6 Nilsson, A., Holme, R., Korte, M., Suttie, N. & Hill, M. Reconstructing Holocene geomagnetic field variation: new methods, models and implications. *Geophysical Journal International* **198**, 229-248, doi:10.1093/gji/ggu120 (2014).

7 Jaffey, A. H., Flynn, K. F., Glendenin, L. E., Bentley, W. C. & Essling, A. M. PRECISION MEASUREMENT OF HALF-LIVES AND SPECIFIC ACTIVITIES OF U-235 AND U-238. *Physical Review C* **4**, 1889-+, doi:10.1103/PhysRevC.4.1889 (1971).

8 Cheng, H. *et al*. Improvements in Th-230 dating, Th-230 and U-234 half-life values, and U-Th isotopic measurements by multi-collector inductively coupled plasma mass spectrometry. *Earth and Planetary Science Letters* **371**, 82-91, doi:10.1016/j.epsl.2013.04.006 (2013).
