# Supplementary material for: Stalagmite paleomagnetic record of a quiet mid-to-late Holocene field activity in central South America
Source: Nat Commun. 2022 Mar 15;13:1349. doi: 10.1038/s41467-022-28972-8 (PMC8924270; doi:10.1038/s41467-022-28972-8)
Supplement: Supplementary file 3 — Description of Additional Supplementary Files [file 41467_2022_28972_MOESM3_ESM.pdf]

Description of Additional Supplementary Files

File Name: Supplementary Data 1
Description: Paleomagnetic dataset obtained

File Name: Supplementary Data 2
Description: **U-Th datapoints**
